# Magnetically Induced Temporal Interference for Focal and Deep-Brain Stimulation

**DOI:** 10.3389/fnhum.2021.693207

**Published:** 2021-09-27

**Authors:** Zonghao Xin, Akihiro Kuwahata, Shuang Liu, Masaki Sekino

**Affiliations:** ^1^Laboratory Sekino, Department of Bioengineering, School of Engineering, The University of Tokyo, Tokyo, Japan; ^2^Laboratory Sekino, Department of Electrical Engineering and Information Systems, Graduate School of Engineering, The University of Tokyo, Tokyo, Japan

**Keywords:** transcranial magnetic stimulation, temporal interference stimulation, temporal interference magnetic stimulation, depth-focality trade-off, coil array

## Abstract

Transcranial magnetic stimulation (TMS) is a non-invasive brain stimulation technique that has been clinically applied for neural modulation. Conventional TMS systems are restricted by the trade-off between depth penetration and the focality of the induced electric field. In this study, we integrated the concept of temporal interference (TI) stimulation, which has been demonstrated as a non-invasive deep-brain stimulation method, with magnetic stimulation in a four-coil configuration. The attenuation depth and spread of the electric field were obtained by performing numerical simulation. Consequently, the proposed temporally interfered magnetic stimulation scheme was demonstrated to be capable of stimulating deeper regions of the brain model while maintaining a relatively narrow spread of the electric field, in comparison to conventional TMS systems. These results demonstrate that TI magnetic stimulation could be a potential candidate to recruit brain regions underneath the cortex. Additionally, by controlling the geometry of the coil array, an analogous relationship between the field depth and focality was observed, in the case of the newly proposed method. The major limitations of the methods, however, would be the considerable intensity and frequency of the input current, followed by the frustration in the thermal management of the hardware.

## 1. Introduction

Transcranial magnetic stimulation (TMS) is a non-invasive brain stimulation method that has been extensively utilized in investigative research and therapeutic applications. As a Food and Drug Administration approved technique, TMS is a potentially effective treatment method for a series of psychiatric disorders, including depressive disorders and Parkinson's disease (Fitzgerald et al., 2006). The theory of TMS is based on the concept that a pulsed magnetic field is delivered to the targeted cortical region via a coil to generate an electric field in the brain as a result of electromagnetic induction. Consequently, the induced electric field can modulate neural activity non-invasively, thus facilitating clinical applications. Unfortunately, because of the rapid attenuation of the magnetic field generated by the TMS coil, TMS is generally unable to stimulate deep brain regions such as ventral striatum and thalamic regions (Deng et al., 2014). This is different from several conventional invasive brain stimulations, such as deep-brain stimulation (DBS). Another crucial factor to achieve the therapeutic effects of TMS is the focality of the induced electric field. This is because the localization of the induced electric field is important to minimize the stimulation of non-target regions, especially when the target region is confined to a restricted area. The stimulation of non-target regions can degrade clinical effectiveness and even cause detrimental side effects (Deng et al., 2014). Considering the aforementioned characteristics of TMS, as well as the substantial interest in stimulating deeper-brain regions, the trade-off between depth and focality could be crucial for the design of TMS coils (Deng et al., 2013).

However, temporal interference (TI) stimulation is a potential non-invasive method that promises stimulation in deep encephalic regions, thus avoiding the overlaying cortex (Grossman et al., 2017). The concept of TI stimulation is that it delivers multiple electric fields with relatively high but slightly different frequencies. Since the mechanisms based on these neural membranes, which possess intrinsic low-pass filtering of electrical signals, have been extensively accepted (Hutcheon and Yarom, 2000), neural electrical activity will not be able to follow very-high-frequency oscillating electric fields. Thus, during TI stimulation, the neurons of the brain do not react directly to these high-frequency electric fields. However, owing to the small frequency difference (e.g., 10 Hz), the two applied fields will consequently form a temporally interfering field, with envelope modulation oscillating at the difference frequency different frequencies that can be followed by the neurons. In conventional TI stimulation methods, electrodes are placed above the scalp to convey the electric current into the brain tissue to penetrate the cranium (Grossman et al., 2017; Sunshine et al., 2021). This has raised concerns that the skin-electrode impedance could deteriorate the performance of the electric current propagation (Zaeimbashi et al., 2020). Consequently, the applied current or voltage could possibly oscillate by maintaining a constant value. Additionally, the asymmetrical geometries of the brain, as well as the tissue impedance distribution, potentially decrease the focality of the electric field generated in the target regions.

Conversely, magnetic stimulation could retain constant voltage and current in the coils, thus ignoring the conductivity properties of the tissue of the subjects. This is mainly attributed to the relatively high electrical impedance of biological tissue, which cannot distort the magnetic field induced by magnetic stimulation (Peterchev et al., 2012). Because of these characteristics of magnetic stimulation, the representative TMS technique implies that the combination of TI stimulation with magnetic stimulation could be an effective approach to realize non-invasive and focal deep-brain stimulation. Herein, we propose a magnetically induced temporal interference stimulation approach utilizing a four-coil configuration to deliver two concurrent sets of interfering electric currents with distinct frequency scales concurrently. A numerical simulation was performed based on a homogeneous hemispherical model, regardless of the non-uniformity of the tissue conductivity. The trade-off between depth and focality was evaluated quantitatively, followed by the comparison of the performance of the proposed scheme with those of several conventional TMS systems.

Notably, considering that the interferential electric field induced in the TI stimulation is essentially oscillating with a constant periodicity, resemble it resembles the Transcranial alternating current stimulation (tACS), which is a non-invasive electrical stimulation method. However, conventional tACS using large electrodes is not as focal as TMS (Antal and Paulus, 2013) due to the distinct stimulation mechanism (i.e., magnetic stimulation vs. electrical stimulation). In addition, the field intensity necessary for a prominent clinical effect differentiates between the two methods. In our study, we also compared TI magnetic stimulation and the tACS.

## 2. Materials and Methods

### 2.1. Brain Model and Induced Electric Field

During the simulation, the human head was modeled using a homogeneous hemisphere model with a radius of 10 cm. Different brain tissue layers were not differentiated so that the model possessed an isotropic inner electrical conductivity of 0.33 S/m, and the cortical surface was assumed to be at a depth of 1.5 cm from the surface of the model. To calculate the electric field induced by high-frequency oscillating currents in the coils, a scalar-potential finite-difference (SPFD) tool developed by our group was employed (Yamamoto et al., 2016). The electric field produced inside the brain model can be acquired by solving the following equation:


(1) $$E=-\frac{\partial A_{0}}{\partial t}-\nabla \phi$$

where **A_0_** is the magnetic vector potential, and ϕ is the scalar potential owing to the accumulation of electrical charge at the interfaces at which conductivity changes.

### 2.2. Proposed Design

**Figure 1:**
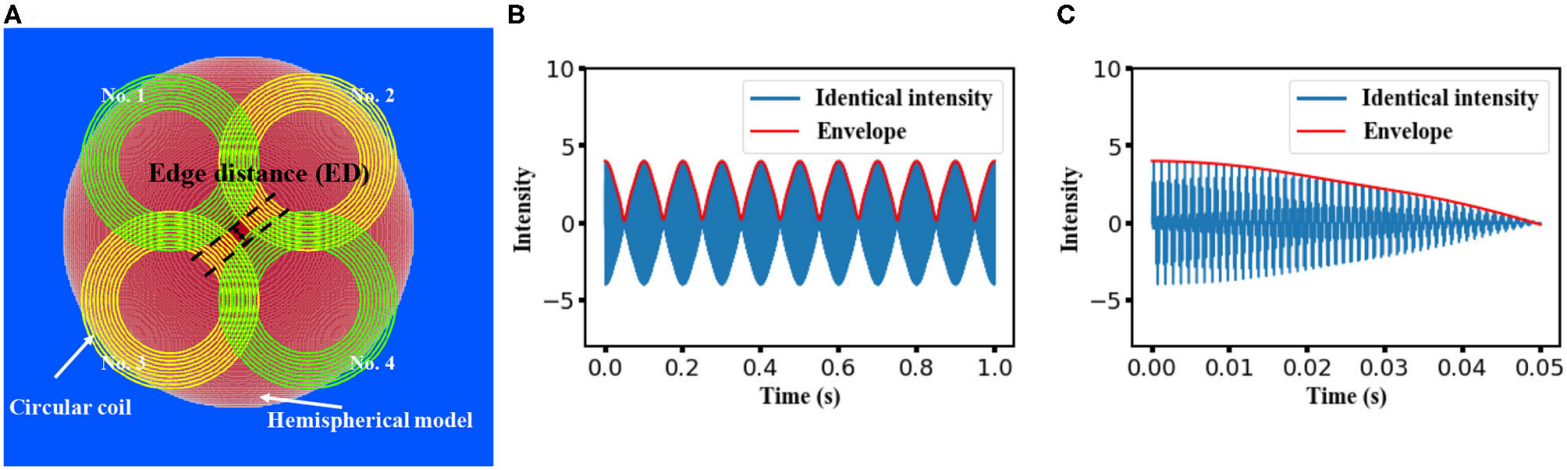
Proposed design for temporal interference magnetic stimulation on a hemispherical model. **(A)** The construction of the stimulation coils and the isotropic head model. A clover-shaped coil array constructed with an inner diameter of 60 mm and an outer diameter of 100 mm. Coils with identical color comprise a “temporal interference set” and are aligned on the same planes at vertical distances of 5 mm and 10 mm above the hemispherical model. **(B)** Feasibility of generating a low-frequency modulation from the four individual waves with a normalized intensity of 1(frequency of 1, 1.01 kHz, 5, and 5.01 kHz, respectively). A time length of 1 s was displayed, and a 10 Hz envelope was observed. **(C)** Same modulation wave with **(B)** but with a time length of 50 ms.

Applying the theory of temporal interference, the temporal interference of high-frequency oscillating electric fields would form an amplitude modulation (envelope) at a low frequency that could be utilized for neural stimulation. The proposed clover-shaped coil configuration used in our study is shown in Figure 1A. Four circular coils composed of 10 wire loops with inner and outer diameters of 60 and 100 mm, respectively, were subdivided into two pairs (coils no. 1 and no. 4 as one pair and coils no. 2 and no. 3 as the other). Each coil pair was symmetrically organized on planes at a vertical distances of 5 and 10 mm above the hemispherical model for coil insulation consideration. The electric current flowing in the two co-planar coils was controlled to achieve a “temporal interference set,” that is, to ensure that the two frequencies were at the kilohertz scale but with a 10 Hz difference. In the proposed design, the frequency was adjusted to 1 and 1.01 kHz in the case of the first pair, while in the case of the other pair, it was set to 5 and 5.01 kHz. Since the intensity of the generated electric field was proportional to both the coil current intensity and frequency, along with the linearity between the scalar potential and the vector potential (Salinas et al., 2009), the magnitude of the current that flowed through the respective coil was inversely proportional to the corresponding frequency.

Notably, coils on different planes could interfere with each other concurrently (e.g., at 1 and 5.01 kHz). Moreover, owing to the existence of harmonics, unexpected low-frequency components could occur in the accumulated electric field generated by the four coils. However, as demonstrated in a previous study (Zhu et al., 2019), the amplitude of this frequency component can be smaller than that of the low-frequency envelope. Herein, we performed simple calculations to account for the superposition of four oscillating waves at the aforementioned frequency. As shown in Figures 1B,C, regardless of the equivalency of the wave amplitude, a modulation with a frequency of 10 Hz could be formed from the four individual waves. This confirmed the feasibility of the stimulation methods introduced in this study.

Moreover, the feasibility of recruiting the neural firing using the four superimposed external stimulus was evaluated based on a simplified single-neuron model. This model consists of conductance of general ion channels (Hodgkin–Huxley mod) and a slow voltage-dependent potassium current, responsible for spike-frequency adaptation, making the model fit to cells from cortex *in vitro* (Pospischil et al., 2008). The neuron response could be described by the following membrane equation


(2) $$C_{m}\frac{\text{d}V}{\;\text{d}t}=-g_{\text{leak }}\left(V-E_{\text{leak }}\right)-I_{\text{Na}}-I_{\text{Kd}}-I_{M}$$

where *V* is the membrane potential, $C_{m}=1mF/cm^{2}$ is leak membrane conductance, *E*_*leak*_ is its reversal potential, and *I*_*ion*_ is determined by respective maximal conductance $\bar{g}$_ion_. Other parameters include neuron dimensions described as a cylinder of diameter *d* and length *L*. The rate constants and the dimensionless quantities in the typical Hodgkin–Huxley model were determined by the spike threshold *V*_*T*_.

### 2.3. Intensity of the Temporal Interfered E-Field

To quantify the intensity of the envelope of the interfered electric field, we initially obtained the electric field distribution in the brain model, which originated from the stimulation coils via the aforementioned simulation methods. The simulation was followed by the computation of the modulation depth in MATLAB (MathWorks, Natick, MA, USA).

The electric field was calculated in three orthogonal directions for each voxel in response to an individual stimulation coil, resulting in a value of **E_i,j,index_** where **i** and **j** represent the coil and voxel number, respectively, while the **index** represents different directions (i.e., x, y, z axis in Cartesian coordinate system).

Subsequently, the depth of the envelope at each voxel was computed along respective directions:


(3) $$\begin{array}{l} \left\|Eenv_{j,index}\right\|=envelope(E_{1,j,index}+E_{2,j,index}+E_{3,j,index}\\ \;+E_{4,j,index}),index=x,y,z, \end{array}$$

which was followed by a computation of the norm of the interfered field vector


(4) $$\left\|Eenv_{j}\right\|=norm\left(Eenv_{j,x},Eenv_{j,y},Eenv_{j,z}\right)$$

Herein, **E**_1…4_ are the respective electric field distributions caused by the electric current with a distinct frequency. It is obvious that the intensity of the envelope reaches a maximum given that the two electric fields have an equivalent magnitude (Huang and Parra, 2019). Herein, we assumed that the target region is located at the central section of the brain model. Since the intensity of the induced electric field was proportional to both the frequency and the current intensity, the applied current intensity was adjusted based on the corresponding frequency (i.e., 1 kA for 1,000 Hz). Currents with different frequencies were applied to each coil simultaneously without phase difference.

### 2.4. Simulation for Conventional TMS Systems

We considered figure-8 TMS coil as a representative choice of TMS systems for its broad applications in therapy (Ueno et al., 1988). In addition to a conventional figure-8 coil, a total of 10 TMS coils were also selected for the simulation and comparison process, referring to the classic review work (Deng et al., 2013), to guarantee the generality of the performance of TMS devices. For all the simulations, TMS coil was placed 5 mm above the surface of the head model for coil insulation consideration, and electric field distribution was obtained by the aforementioned FEM solver with an input current intensity of 1 kA.

### 2.5. Quantification of Trade-Off Between Depth and Focality

To quantitatively evaluate the performance of our proposed magnetically induced TI stimulation method with the conventional TMS stimulation schemes, two crucial characteristics of magnetic stimulation, focality and electric field penetration, were calculated based on our simulation results. The electric field penetration *d*_1/2_[*cm*] was defined as the radial distance from the surface of the cortex to the voxel, where the electric field strength was attenuated to half of the maximum value of the cortical surface. Correspondingly, the half-maximum volume value $V_{1/2}\left[cm^{3}\right]$ can be defined as the number of voxels in which the field intensity exceeds the threshold value (i.e., half of the maximum value) inside the “brain.” Additionally, the focality *S*_1/2_[*cm*] that indicates the spread of the electric field distribution was determined by the ratio of the half-maximum value volume to the *d*_1/2_ (Deng et al., 2013).

### 2.6. Simulation for a Conventional tACS Stimulation

The identical head model was applied for the tACS simulation, and two circle electrodes (radius of 2.8 cm and height of 0.5 cm) were modeled as anode and cathode electrode, respectively, and were placed on the surface of the head with an interval of 45 degrees to the center of the model. An isotropic conductivity value of 2 S/m was assigned to the electrodes and the stimulation intensity was determined as 1 mA, allocated with a frequency of 10 Hz. Electric field distribution was solved with the FEM solver in COMSOL multiphysics (COMSOL, Inc., Burlington, MA, USA).

## 3. Results

**Figure 2:**
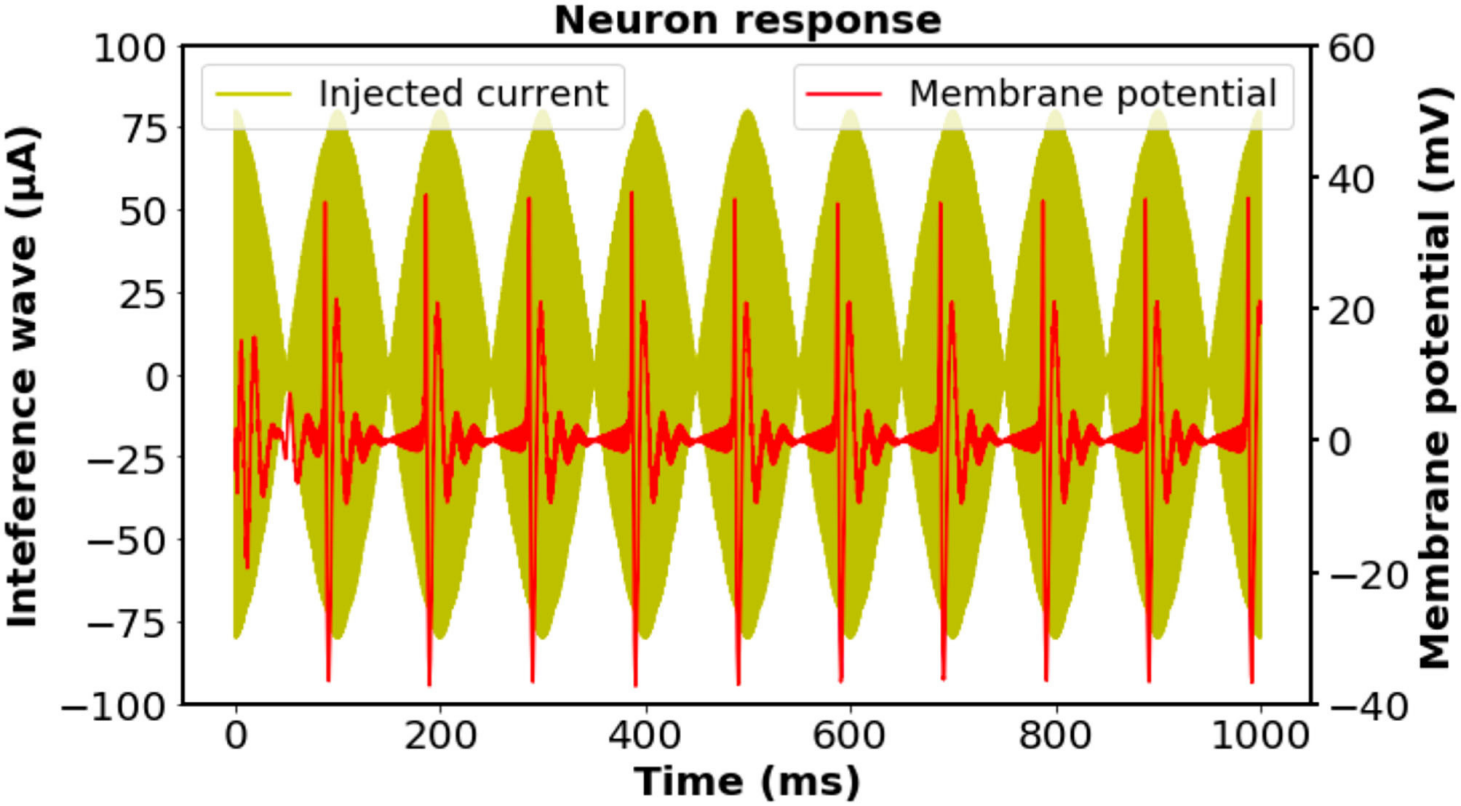
Investigation of the biological neuronal response and the time-matched stimulus at the designed frequency according to the regular-spiking neuron model. The intensity of each external current was set to be the same, while the total current intensity was adjusted to have a reasonable value. The neural response was filtered by a fourth-order Butterworth bandpass filter with cut-off frequencies of 50 and 500 Hz to remove the stimulation artifacts. A 10 Hz oscillation of the membrane potential was observed, which was consistent with the frequency of the interfering stimulation wave. Neuron parameters: length and diameter: *L* = *d* = 61.4μm, leak membrane conductance: $g_{\text{leak}}=2.05\times 10^{-5}\text{S}/\text{c}{\text{m}}^{2}$, reversal potential: *E*_leak_ = −70.3mV, maximal conductance: ${\bar{g}}_{\text{Na}}=0.056\;\text{S}/{\text{cm}}^{2},{\bar{g}}_{\text{Kd}}=0.006\;\text{S}/{\text{cm}}^{2},{\bar{g}}_{M}=7.5\times 10^{-5}\;\text{S}/{\text{cm}}^{2}$. Spike threshold: *V*_*T*_ = −56.2mV.

We initially investigated the feasibility of the proposed stimulation scheme on biological neurons by exploiting a modified Hodgkin–Huxley model; thus, the effectiveness can be certified by the occurrence of neural activation at the corresponding frequency (Cao and Grover, 2019). Notwithstanding the existence of the directional sensitivity of the neural excitation against stimulation (Aberra et al., 2020), for the sake of simplicity, herein we assumed that the axon of the neuron is oriented parallel to a homogeneous electric field. The stimulation on the neuron was assumed to be the imposition of four injection currents with the designed frequency simultaneously. The intensity of each external current was set to be the same, while the total current intensity was accommodated to a reasonable value for inducing neural activation. As illustrated in Figure 2, a 10-Hz neural response was observed in accordance with the oscillation of the 10-Hz modulation envelope produced by the four individual input currents, indicating that the neurons could be driven by the interference electric field generated from the four coils.

**Figure 3:**
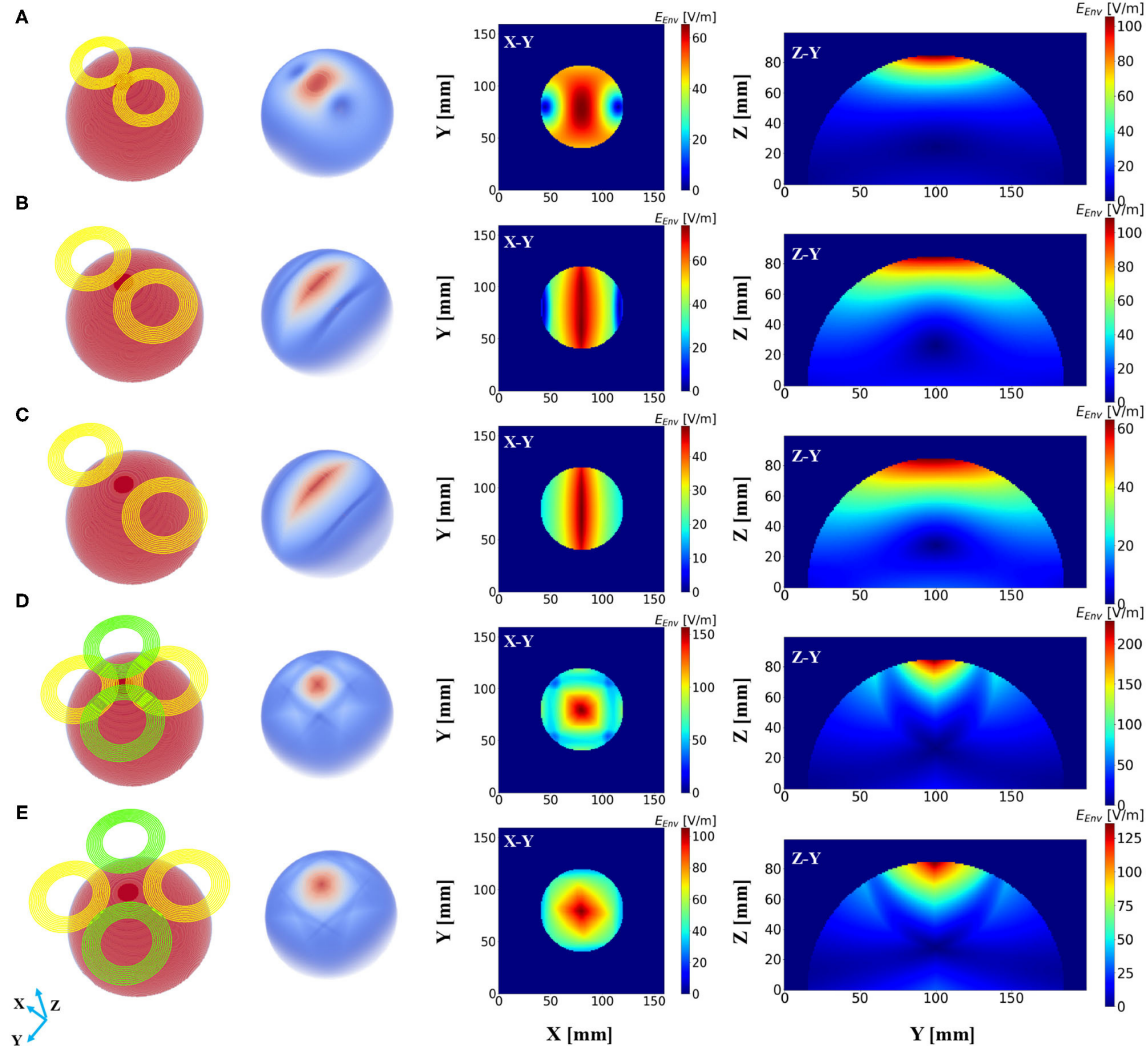
Electric field distribution in the hemispherical model induced by distinct stimulation protocols. The first column shows the geometry of the stimulation coils, and the second column shows the overall dispersal of the inferential field. Cross-sections of the model from different direction axes are displayed in the last two columns. The current intensity in all the coils was determined to be 1 kA. **(A)** Magstim 70 mm figure-8 coil (P/N 9925, 3190). **(B)** Two-coil temporal interference magnetic stimulation with an edge distance of 10 mm. **(C)** Two-coil temporal interference magnetic stimulation with an edge distance of 40 mm. **(D)** Four-coil temporal interference magnetic stimulation with an edge distance of 10 mm. **(E)** Four-coil temporal interference magnetic stimulation with an edge distance of 40 mm.

Subsequently, a field distribution map was depicted to evaluate the performance of different stimulation protocols based on the consideration of the depth at which the stimulation effect could reach and the spread of the induced field. Figure 3A demonstrates the distribution of the induced electric field distribution in the hemispherical brain model activated by a conventional figure-8 (Fitzgerald et al., 2006) TMS coil (Magstim 70 mm, figure-8 coil [P/N 9925, 3190]). Cross-sections of the model from different directional axes are displayed in the figure. The figure-8 TMS coil was introduced three decades ago to enhance the focality of the TMS systems (Ueno et al., 1988). Currently, the figure-8 shape coil is considered one the most efficient choices for constructing commercial TMS systems. Unsurprisingly, the substantially stimulated regions were mainly distributed in the superficial area of the model; these correspond to the cortical regions in a realistic brain model. The electric field penetration of this figure-8 coil was 1.4 cm with a focality of 14.67 *cm*^2^. These findings are similar to those reported previously (Deng et al., 2014). Nevertheless, a total field over 100 V/m is necessary to stimulate the neurons in a classical or repetitive TMS therapy (Grehl et al., 2016), and our numerical computation results showed that the field intensity overwhelmingly surpassed this value, which could cause safety concerns. This can be addressed by optimizing the intensity of the current that flows through the coils. However, in a clinical application, the current intensity should be rigorously determined, as it is done in a conventional TMS (determining the Motor threshold) or tACS (intracranial measurements) therapy (Rossini et al., 2015; Huang et al., 2017).

We then conducted the simulation, which employed only two circular coils (i.e., analogous to a figure-8 TMS coil) to realize TI magnetic stimulation referring to the results from previous studies (Sorkhabi et al., 2020), considering the uncomplicated structure of the coil configuration. The edge distance (ED) between the two coils was initially set at 0 mm and then increased to 40 mm with an interval of 5 mm. Figures 3B,C show the resulting electric field map with EDs of 10 and 40 mm, respectively. Furthermore, we observed an oval-shaped electric field distribution (X-Y planar) that differs from the previously reported results (Sorkhabi et al., 2020). This might be attributed to the distinct model geometry used for evaluation, given that the asymmetry of the tissue geometry can dramatically affect the concentration of the interferential electric field. Furthermore, we realized that the increase in the stimulation depth, which was indicated by the value of *d*_1/2_, was accompanied by reduced focality. This is similar to conventional TMS systems as described above. Nevertheless, this is inconsistent with the findings of previous reports, where the results demonstrated the stability of the field dispersion along with the variation in the field penetration (Sorkhabi et al., 2020).

**Figure 4:**
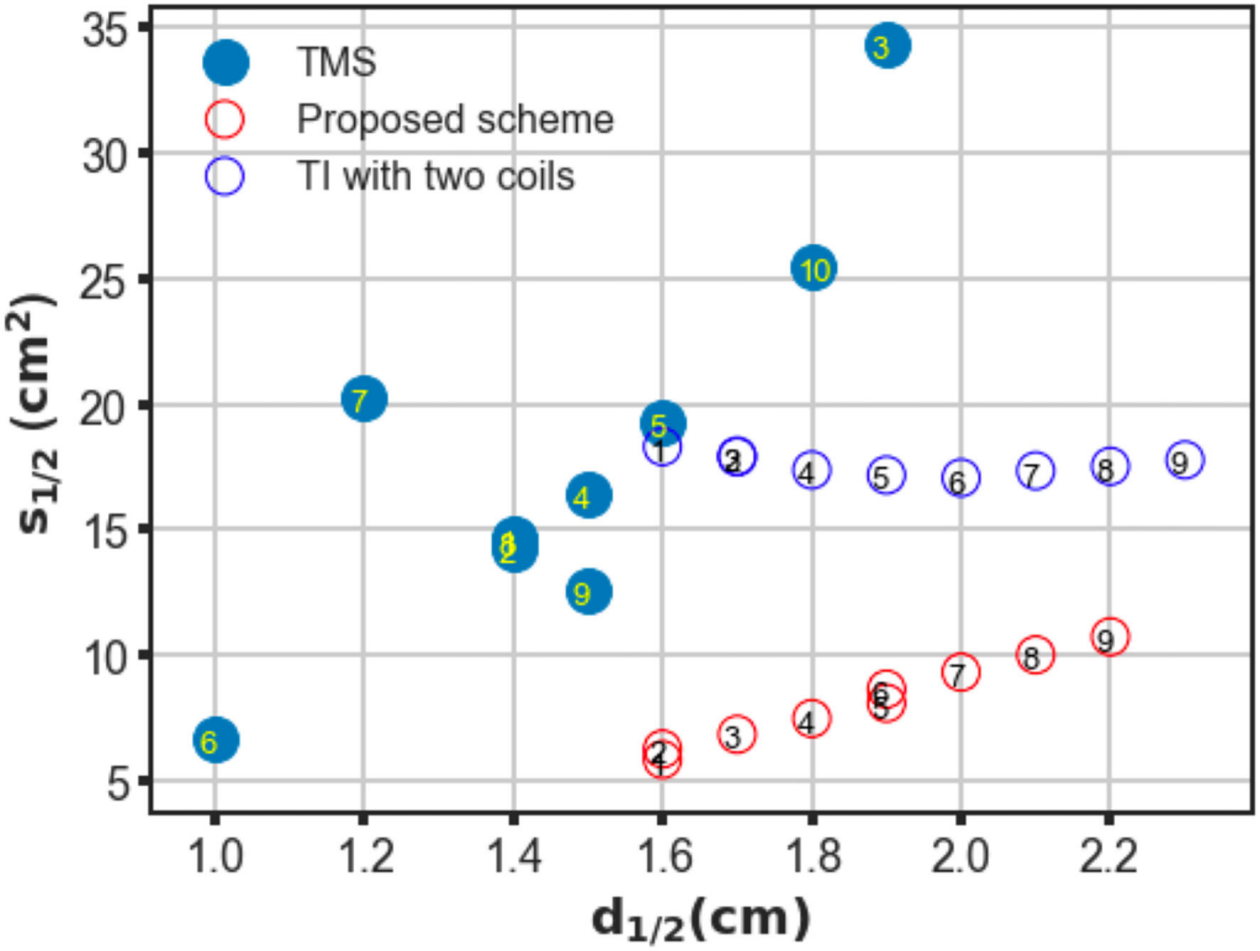
Trade-off between depth (*d*_1/2_) and focality (*S*_1/2_) of conventional TMS systems, two-coil, and four-coil TI stimulations. For TMS systems, different coil configurations are labeled as follows: ① Magstim 70 mm figure-8 coil (P/N 9925, 3190), ② MagVenture C-B65 butterfly coil, ③ Magstim double cone (P/N 9902), ④ MagVenture MC-B70 butterfly, ⑤ Eccentric double cone coil with center-dense windings, ⑥ Magstim 25 mm figure-8 (P/N 1165), ⑦ Cadwell Corticoil, ⑧ Cadwell B-shaped coil, ⑨ 50 mm V-coil, and ⑩ MagVenture D-B80 For the TI magnetic stimulation coils, the edge distance between two opposite coils was changed from 0 to 40 mm at 5 mm intervals (1–9).

The proposed scheme was evaluated in an identical process to obtain the correlation between two critical indices. In contrast with the figure-8 coil and two-coil TI magnetic stimulation, we demonstrated an increase in *d*_1/2_ and a decrease in the value of *S*_1/2_ of the induced low-frequency envelope based on the utilization of our proposed TI magnetic stimulation scheme, thus indicating the improvement of the stimulation depth and focality. As shown in Figure 3D, by setting the distance between two confronting coils in the same plane to 10 mm, the *d*_1/2_ and *S*_1/2_ values of the interfered electric field were 1.7 cm and 6.87 *cm*^2^, respectively. From the results, the superiority of the designed method to the figure-8 TMS coil is displayed by the fact that the limited depth to which the electric field can be delivered to by the TMS coil, was considered. This performance also surpasses the two-coil TI magnetic stimulation owing to the confined field dispersion, whereas the field induced by the two coils was capable of reaching a depth similar to that of the model. In the case wherein the ED was chosen to be 10 mm, the *d*_1/2_ values of the two modalities achieved comparable values of 1.7 cm (four coils) and 1.7 cm (two coils); however, the *S*_1/2_ value decreased by 61% when the introduced clover-shaped coils were utilized. Similarly, to systematically investigate the stimulation effects of the proposed methods, simulations were conducted by increasing the distance between two confronting coils (ED) from 0 to 40 mm with an equal interval of 5 mm (Figure 3E shows the case wherein the ED was set at 40 mm). By depicting the distribution of the depth focality of coils with discrete ED, as illustrated in Figure 4, we observed an analogous tendency with TMS systems. That is, at increasing ED, the *d*_1/2_ increased, notwithstanding the *S*_1/2_ diminished proportionally. This is attributed to the property of TI stimulation, which could be mathematically demonstrated by the fact that the modulation depth of the interfered waveform cannot exceed the sum of the magnitudes of the individual signals. Consequently, the distribution of the low-frequency envelope was determined by the electric field induced by the respective coil, which was essentially equivalent to the magnetic stimulation. Therefore, there exists a trade-off between the field penetration and the field spread in TI magnetic stimulation method. This is in accordance with our suggested scheme, which promised stimulation in relatively deep regions of the brain and the concurrent constraint of the spreading of the electric field.

To confirm the superiority of the proposed scheme against conventional TMS systems, a series of conventional TMS coils, some of which are commercial products, were employed in the simulations for additional comparisons. These coils included figure-8 coils of symmetric and asymmetric structures with distinct coil geometry, butterfly coils, and double cone coils formed by two circular coils fixed at an angle (Deng et al., 2013). The trade-off between depth and focality was computed for all coils and plotted in Figure 4. As shown in the figure, in TMS systems, coils provided deeper field penetration and could not retain the concentration of the induced electric field. Conversely, coil types that realized localized stimulation failed to propagate the electric field in deep model regions. However, using TI magnetic stimulation method, we demonstrated a potential solution to overcome the limitation of the depth-focality trade-off in conventional TMS systems. The maximum electric field intensity for each coil configuration was summarized in the Table 1.

**Table 1:** Maximum electric field intensity of different coil design (V/m).

	**①**	**②**	**③**	**④**	**⑤**	**⑥**	**⑦**	**⑧**	**⑨**	**⑩**
TMS coils	273.34	217.65	123.01	128.28	507.8	288.40	293.37	415.01	137.14	195.73
TI with two coils	276.15	235.81	201.67	173.59	150.45	131.20	115.05	101.37	89.69
Proposed design	523.93	485.26	411.95	348.82	296.84	274.07	238.29	208.18	182.18

**Figure 5:**
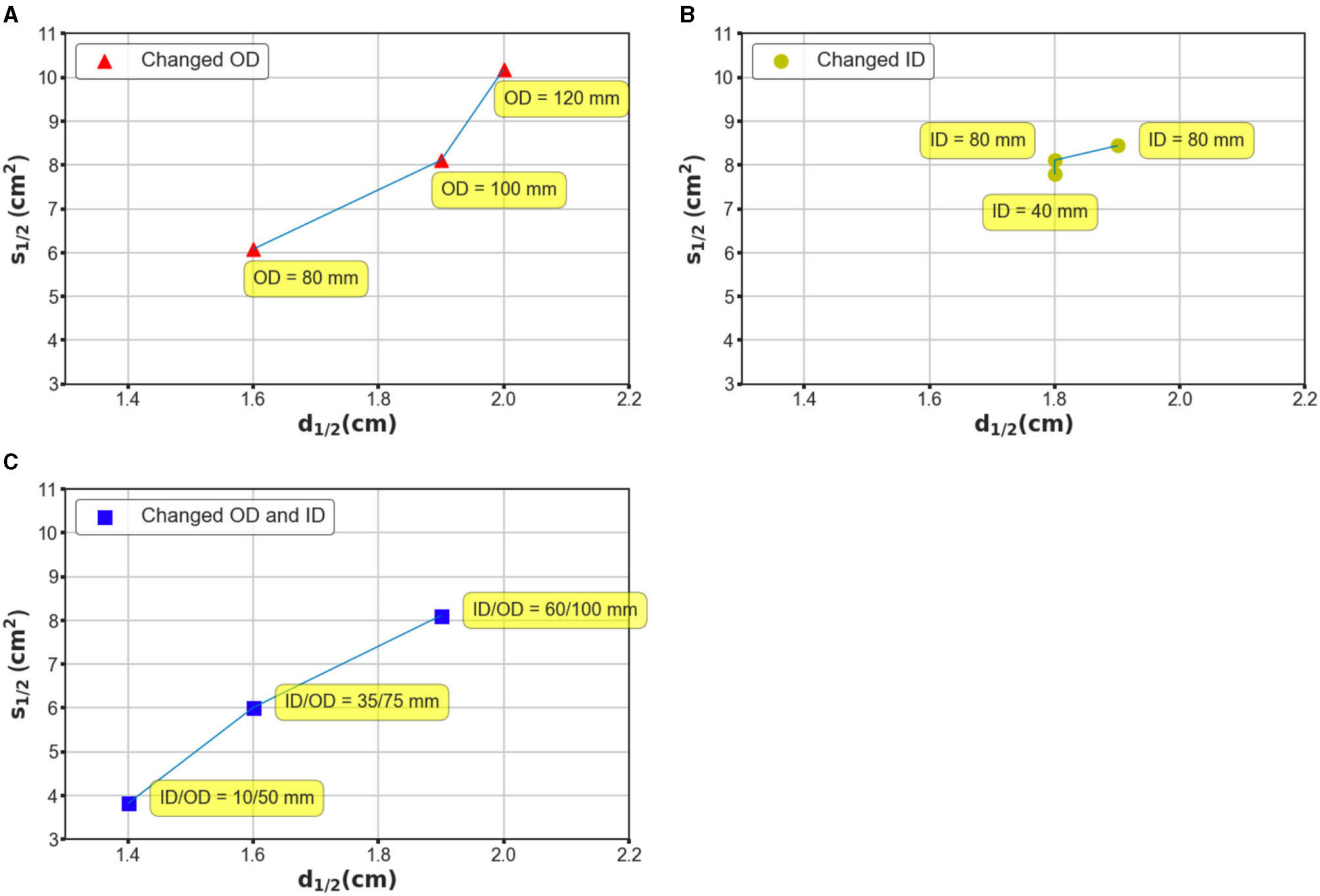
Investigation of mechanism based on which the coil geometry can affect the performance of the stimulation. **(A)** The OD was designated as 80, 100, and 120 mm, while the ID was maintained at 80 mm. **(B)** The ID was designated as 80, 60, and 40 mm, while the OD was maintained at 100 mm. **(C)** The OD and ID were changed simultaneously such that the interval remained constant. The OD–ID values are 10–50, 35–75, and 60–100 mm, respectively.

Additionally, coil geometry can drastically affect the field distribution; therefore, we recognized the necessity of evaluating the stimulation effects with different coil parameters. We conducted three batches of simulations based on considerations of two general geometry parameters, namely the ID and OD. In the case of the first batch, the OD was set at 80, 100, and 120 mm, while the ID was maintained at 60 mm; in the case of the second batch, the ID changed from 60 to 40 mm and 80 mm, and the OD was retained at 100 mm. Finally, the OD and ID were changed simultaneously such that the interval remained constant. Figure 5 shows the field distributions by exploiting ultra-small coil arrays (Figure 5A, ID/OD = 10/50 mm) and small dimension coil arrays (Figure 5B, ID/OD = 35/75 mm). As illustrated in Figure 5, by enlarging the OD, the electric field tends to be more widespread along the tangential and radial orientations (Figure 5A), and it covered large regions of the hemispherical model. The ID increment, however, promised a more restricted field distribution (Figure 5B). This tendency suggests that adjusting OD rather than ID appears to ensure a greater impact, which can be intuitively defined by the variation in *S*_1/2_ over the same disparity of *d*_1/2_ on the generated field. This tendency is repeatedly confirmed by the simulation results using coils with identical widths but different OD/IDs (Figure 5C). Coils with large dimensions are capable of delivering an electric field to an extended area, which is expected to be employed for stimulation targets deeper than the cortex.

**Figure 6:**
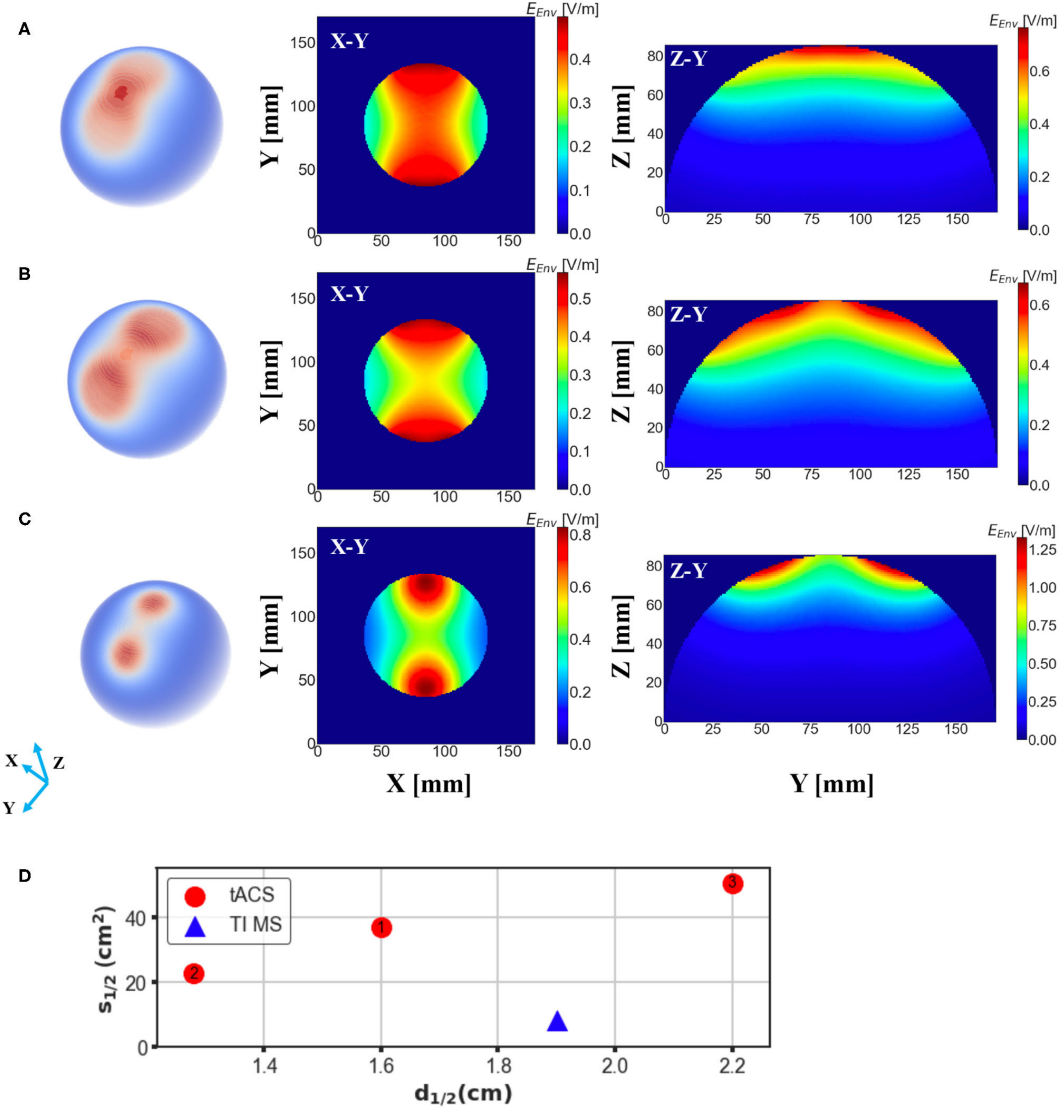
Electric field distribution in the head model induced by a conventional tACS scheme with distinct electrode montages. E-field spread on the surface of the brain is shown in the first column and cross-sections of the model (brain part) from different direction axes are displayed in the last two columns. **(A)** Two electrodes with a radius of 2.8 cm were placed symmetrically on the surface of the head with an interval of 45 degree to the center. **(B)** The same setup as **(A)** but the angular separation was changed to 60 degree. **(C)** The same setup as (A) but the radius of the electrodes was changed to 1.4 cm. **(D)** Depth-focality performance of the tACS in comparison with the suggested TI magnetic stimulation scheme (ED 20 mm). TI magnetic stimulation exhibits better performance for a focal and relatively deep stimulation.

Finally, we investigated the electric field distribution from a conventional tACS paradigm by applying the analogous quantitative calculation. Notably, the essential induced electric field, maximum value of which in our simulation reached to 0.76 V/m, is much smaller than that in either conventional TMS systems or the proposed design, which is mainly owing to the substantial coil current intensity. Our simulation results showed that the stimulation depth of the tACS is comparable to the magnetic stimulation methods with a value of 1.6 cm (Figure 6A); however, the spread of the electric field was much larger than TI magnetic stimulation resulting in a spread area *S*_1/2_ of 36.87 *cm*^2^. The focality of the tACS could be even worse accompanied with the increment of electrode interval, as shown in Figure 6B, when the interval angel between two electrodes was set at 60 degrees, the consequent spread area reached 50.45 *cm*^2^, meaning the tACS sacrificed focality for a deeper stimulation effect. Moreover, the focality of conventional tACS could be enhanced by reducing the electrode scale, which unfortunately adversely diminishes the penetration depth adversely. Figure 6C demonstrates that when the radius of the electrodes was reduced by 1/2 (1/4 area), the stimulation area could be much more focal but the value of *d*_1/2_ decreased to 1.28 cm consequently. Thus, our proposed scheme displays superior results (Figure 6D).

## 4. Discussion

In conventional TMS systems, the stimulation effects are restrained by the depth and focality trade-off of the induced electric field. Typically, coils with larger dimensions produce electric fields with greater *d*_1/2_ and *S*_1/2_, whereas smaller coils produce electric fields that are more localized and superficial (Deng et al., 2014). Therefore, TMS systems are not invariably applicable when clinical interest is considered to stimulate brain regions deeper than the superficial cortex. This is because the spread of activation of non-target brain regions may deteriorate the expected clinical outcomes or even cause severe side effects.

Herein, we designed a scheme that performs a temporally interfered magnetic stimulation utilizing a four-coil structure. We observed that our proposed study manages to convey the stimuli to deeper regions of the brain model by exploiting four sinusoidal fields that produce oscillations at the frequency of 10 Hz. It should be noted that although previous studies have reported an analogous design, wherein multiple electrodes carrying distinct frequencies were exploited, their aim was the realization of multipoint stimulation (Zhu et al., 2019). By contrast, our study is more interested in the exploration of the feasibility of the production of localized single-point stimulation. However, one critical issue to be addressed in both studies is the selection of the appropriate current frequency owing to the existence of frequency harmonics. The harmonic components of the low-frequency field, interfere with the high-frequency field and therefore generate an envelope with an undesirable frequency, thus distorting the envelope of the target frequency. One solution to this question, as illustrated by the same study, is to increase the differential frequency between the low-and high-frequency field waves such that the amplitude ratio of the unexpected modulation to the primary component can be significantly diminished.

Numerical simulations were then implemented for TI magnetic stimulation and TMS systems, and the relationship between field penetration and focality were computed quantitatively. A wide range of different types of TMS were explored herein that provided systematic insight into the limitations of existing TMS systems. In comparison with TMS systems, TI magnetic stimulation promised a larger *d*_1/2_, while *S*_1/2_ was maintained at a small value that may be considered a breakthrough for overcoming the limitations of conventional TMS systems. Additionally, the integration of the concept of magnetic stimulation in TI stimulation instead of using electrodes guarantees the stability of the applied currents, thus ensuring a more controllable manipulation of the entire system. It should be noted that in this study, Double cone TMS coil is generally considered applicable to a deep TMS stimulation (Lu and Ueno, 2017), targets of which could be positioned at brain regions underneath the cortical area (e.g., subcortex), whereas based on our simulation, the suggested TI magnetic stimulation could possibly reach farther from the cortical surface compared to Double cone coil, i.e., with a potential of increasing the clinical therapeutic (e.g., antidepressant) effect (Lu and Ueno, 2017). Nevertheless, a depth of 2–3 cm seems to be the limit of our results. This could be due to the choice of the model that stimulation depth of Double cone coil could reach 3–4 cm in realistic brain model (while 2 cm in sphere model) or clinical cases (Lu and Ueno, 2017). We argue that the suggested method could possibly reach a depth of over 4 cm when applied to a realistic brain model.

Considering the coil configuration for TI magnetic stimulation, the application of two coils (analogous to the figure-8 TMS coil) appears to be more straightforward. However, there are concerns that the induced field cannot distribute anisotropically. As indicated by our simulation, localized activation was ensured along the orientation of the center-line, whereas, in the orthogonal orientation to this axis, a broad range of the area was stimulated. However, by performing the stimulation using the four-coil configuration, we addressed the spread of the induced electric field which derived a relatively concentrated stimulation area accompanied by a beneficial field penetration.

Notably, even in TI magnetic stimulation, the stimulation effects could be affected considerably by the design of the coils, similar to that in TMS systems. We also found that it would be impractical to suppress the loss of focality, while improving the stimulation depth unilaterally. This depth focality could be determined by several crucial factors of the coil geometry, such as the edge distance, which was demonstrated by our simulations. In addition, we explored a range of coil design parameters that comprised different OD and ID values. The application of considerably larger coils ensures an electric field with larger penetration but conversely has a non-focal field distribution. Interestingly, using a coil with a comparatively small dimension (ID–OD of 10–50 mm), the electric field spread can be significantly suppressed with an *S*_1/2_ value of 3.82 *cm*^2^ (Figure 5A). This configuration still guarantees a *d*_1/2_ value of 1.4 cm, which is equivalent to a conventional figure-8 TMS coil. While the proposed TI magnetic stimulation is aimed to stimulate the target underneath the cortical regions, the ultra-small coils provided us with a potential candidate for highly concentrated, superficial brain stimulation. Herein, we demonstrated how the coil geometry can affect the induced interfering field even by modifying the basic parameters of the coil structure. Since coil optimization methods have been extensively discussed in TMS research studies;, future work could focus on the optimization of the coil design using numerical and experimental methods for a more efficient stimulation (Koponen et al., 2017; Gomez et al., 2018).

Since this study mainly focused on magnetic stimulation methods, we also performed an uncomplicated simulation of electric stimulation for comparison. tACS is an electrical stimulation method based on the application of periodically oscillating electrical currents, which is similar to the application of TI electrical stimulation (Rampersad et al., 2019). However the conventional tACS method is limited by the less lower focality in comparison with magnetic stimulation methods (Antal and Paulus, 2013; Herrmann et al., 2013), which was also demonstrated by our simulation. Notably, though the spread of the E-field could be slightly suppressed by adjusting the electrodes alignment and montage, the depth-focality trade-off of tACS was still outperformed by TI magnetic stimulation, especially considering the large non-target areas that are potentially exposed to the electric field induced by tACS. Since this study aims to develop a focal and deeper brain stimulation scheme, leveraging the characteristics of the magnetic stimulation, only a basic comparison was conducted exploiting a simple tACS scheme without considering other complicated tACS montages (Datta et al., 2009; Karabanov et al., 2019).

Although a substantial advance over prior work was demonstrated, several limitations of the model should be noted. In this study, we assessed the neural response to a stimulus composed of four frequency components. Nevertheless, general concerns still exist on whether high-intensity electric fields at frequencies of the order of kilohertz can substantially deteriorate the neuronal activation (Grossman et al., 2017). These should be examined rigorously, especially for prospective clinical use. Some previous studies have explored the high-frequency conduction block biophysics of TI stimulation (Mirzakhalili et al., 2020), while others have investigated the membrane characteristics in hippocampal gamma oscillations attributed to interfering stimulation (Esmaeilpour et al., 2021). In our simulation, the envelope frequency was determined as 10 Hz, which is a suitable choice for both computational and experimental study (Grossman et al., 2017). However, this modulation frequency should also be determined according to the anticipated clinical effects, for example, 5Hz, to modulate the gamma oscillation (Esmaeilpour et al., 2021). Therefore, future work could focus on the investigation of the mechanisms of brain activation in response to TI neuronal stimulation in both animal models and realistic clinical situations. In addition, one limitation existing in the study is that a homogeneous hemispherical model was employed for the simulation, as we focused on evaluating the performance of both TMS and TI magnetic stimulation in a general condition, without considering the individual difference that could be brought using a realistic human or animal model (Eaton, 1992; Deng et al., 2013). However, this could possibly introduce a bias in the clinical applications, which should be eliminated by a pre-therapeutic estimation for each individual.

Another limitation is the tediousness of the realistic design of the system. We primarily focused on evaluating the electric field distribution of TI magnetic stimulation as a potential alternative for TMS and, therefore, exploited identical current intensity with TMS. Consequently, thermal management and energy efficiency are non-trivial regarding the considerably large current intensity and frequency, which may raise concerns regarding hardware restrictions. These issues were discussed in published TMS research studies (Rossi et al., 2009; Deng et al., 2014). These could become more essential for TI magnetic stimulation regarding the necessity of administration of continuous high-intensity, as well as and high-frequency currents through the coils.

It should be noted that the coil current intensity employed for our simulation was 1,000 A, which could be impractical for fabrication purposes. Even if we accommodate this value to the minimum E-field limitation in reference to TMS systems, the thermal problems are still non-negligible. We performed a simple calculation, assuming a typical TMS copper coil with an area of 7 *mm*^2^, length of 2.5 m, and applied current intensity of 500 A: the resistance of the total wire would be $\text{R}=\text{L}/\sigma_{\text{copper}}/\text{Area}\approx 6.05\times 10^{-3}\Omega$, then the resistance power would be P = R × rms(I)^2^ ≈ 0.756kW, followed by a temperature rising rate of P/(M_coil_ × c_copper_) ≈ 12.5K/s, at which the coil could be possibly destructed in a short period. However, as we discussed above, the stimulation waveform of the TMS and TI magnetic stimulation is distinct, where TMS delivers brief bursts of about 100–300 μs duration, TI generates continuous oscillating fields at the modulation frequency analogous to tACS. We argue that this distinction in the stimulation form could lead to inconsistent neuron physiological mechanisms underlying the stimulation effects, thus TI magnetic stimulating is more likely to modulate spontaneous firing rates by a low intensity field as than the tACS dose (Herrmann et al., 2013; Rossini et al., 2015). If so, the coil current intensity could be potentially restricted in a relatively small value that is feasible for a practical application and consequently, the heating issue of the coil could be addressed by introducing the cooling system as done by TMS devices (Parthoens et al., 2016).

## Data Availability Statement

The original contributions presented in the study are included in the article/supplementary material, further inquiries can be directed to the corresponding author/s.

## Author Contributions

ZX, AK, SL, and MS designed the research. ZX performed the research, analyzed the data, and wrote the paper. ZX, AK, and SL discussed the interpretation of the results. All authors revised the final manuscript.

## Conflict of Interest

The authors declare that the research was conducted in the absence of any commercial or financial relationships that could be construed as a potential conflict of interest.

## Publisher's Note

All claims expressed in this article are solely those of the authors and do not necessarily represent those of their affiliated organizations, or those of the publisher, the editors and the reviewers. Any product that may be evaluated in this article, or claim that may be made by its manufacturer, is not guaranteed or endorsed by the publisher.

## References

[B1] Aberra A. S. Wang B. Grill W. M. Peterchev A. V. (2020). Simulation of transcranial magnetic stimulation in head model with morphologically-realistic cortical neurons. Brain Stimul. 13, 175–189. 10.1016/j.brs.2019.10.002 31611014 PMC6889021

[B2] Antal A. Paulus W. (2013). Transcranial alternating current stimulation (tACS). Front. Hum. Neurosci. 7:317. 10.3389/fnhum.2013.00317 23825454 PMC3695369

[B3] Cao J. Grover P. (2019). Stimulus: noninvasive dynamic patterns of neurostimulation using spatio-temporal interference. IEEE Trans. Biomed. Eng. 67, 726–737. 10.1109/TBME.2019.2919912 31150335

[B4] Datta A. Bansal V. Diaz J. Patel J. Reato D. Bikson M. (2009). Gyri-precise head model of transcranial direct current stimulation: improved spatial focality using a ring electrode versus conventional rectangular pad. Brain Stimul. 2, 201–207. 10.1016/j.brs.2009.03.005 20648973 PMC2790295

[B5] Deng Z.-D. Lisanby S. H. Peterchev A. V. (2013). Electric field depth-focality tradeoff in transcranial magnetic stimulation: simulation comparison of 50 coil designs. Brain Stimul. 6, 1–13. 10.1016/j.brs.2012.02.005 22483681 PMC3568257

[B6] Deng Z.-D. Lisanby S. H. Peterchev A. V. (2014). Coil design considerations for deep transcranial magnetic stimulation. Clin. Neurophysiol. 125, 1202–1212. 10.1016/j.clinph.2013.11.038 24411523 PMC4020988

[B7] Eaton H. (1992). Electric field induced in a spherical volume conductor from arbitrary coils: application to magnetic stimulation and MEG. Med. Biol. Eng. Comput. 30, 433–440. 10.1007/BF02446182 1487945

[B8] Esmaeilpour Z. Kronberg G. Reato D. Parra L. C. Bikson M. (2021). Temporal interference stimulation targets deep brain regions by modulating neural oscillations. Brain Stimul. 14, 55–65. 10.1016/j.brs.2020.11.007 33186778 PMC9382891

[B9] Fitzgerald P. B. Fountain S. Daskalakis Z. J. (2006). A comprehensive review of the effects of RTMS on motor cortical excitability and inhibition. Clin. Neurophysiol. 117, 2584–2596. 10.1016/j.clinph.2006.06.712 16890483

[B10] Gomez L. J. Goetz S. M. Peterchev A. V. (2018). Design of transcranial magnetic stimulation coils with optimal trade-off between depth, focality, and energy. J. Neural Eng. 15:046033. 10.1088/1741-2552/aac967 29855433 PMC6433395

[B11] Grehl S. Martina D. Goyenvalle C. Deng Z.-D. Rodger J. Sherrard R. M. (2016). In vitro magnetic stimulation: a simple stimulation device to deliver defined low intensity electromagnetic fields. Front. Neural Circ. 10:85. 10.3389/fncir.2016.00085 27857683 PMC5093126

[B12] Grossman N. Bono D. Dedic N. Kodandaramaiah S. B. Rudenko A. Suk H. J.. (2017). Noninvasive deep brain stimulation via temporally interfering electric fields. Cell 169, 1029.e16–1041.e16. 10.1016/j.cell.2017.05.024 28575667 PMC5520675

[B13] Herrmann C. S. Rach S. Neuling T. Strüber D. (2013). Transcranial alternating current stimulation: a review of the underlying mechanisms and modulation of cognitive processes. Front. Hum. Neurosci. 7:279. 10.3389/fnhum.2013.00279 23785325 PMC3682121

[B14] Huang Y. Liu A. A. Lafon B. Friedman D. Dayan M. Wang X.. (2017). Measurements and models of electric fields in the *in vivo* human brain during transcranial electric stimulation. eLife 6:e18834. 10.7554/eLife.18834 28169833 PMC5370189

[B15] Huang Y. Parra L. C. (2019). Can transcranial electric stimulation with multiple electrodes reach deep targets? Brain Stimul. 12, 30–40. 10.1016/j.brs.2018.09.010 30297323 PMC6301116

[B16] Hutcheon B. Yarom Y. (2000). Resonance, oscillation and the intrinsic frequency preferences of neurons. Trends Neurosci. 23, 216–222. 10.1016/S0166-2236(00)01547-2 10782127

[B17] Karabanov A. N. Saturnino G. B. Thielscher A. Siebner H. R. (2019). Can transcranial electrical stimulation localize brain function? Front. Psychol. 10:213. 10.3389/fpsyg.2019.00213 30837911 PMC6389710

[B18] Koponen L. M. Nieminen J. O. Mutanen T. P. Stenroos M. Ilmoniemi R. J. (2017). Coil optimisation for transcranial magnetic stimulation in realistic head geometry. Brain Stimul. 10, 795–805. 10.1016/j.brs.2017.04.001 28461068

[B19] Lu M. Ueno S. (2017). Comparison of the induced fields using different coil configurations during deep transcranial magnetic stimulation. PLoS ONE 12:e0178422. 10.1371/journal.pone.0178422 28586349 PMC5460812

[B20] Mirzakhalili E. Barra B. Capogrosso M. Lempka S. F. (2020). Biophysics of temporal interference stimulation. Cell Syst. 11, 557–572. 10.1016/j.cels.2020.10.004 33157010

[B21] Parthoens J. Verhaeghe J. Servaes S. Miranda A. Stroobants S. Staelens S. (2016). Performance characterization of an actively cooled repetitive transcranial magnetic stimulation coil for the rat. Neuromodul. Technol. Neural Interface 19, 459–468. 10.1111/ner.12387 26846605

[B22] Peterchev A. V. Wagner T. A. Miranda P. C. Nitsche M. A. Paulus W. Lisanby S. H.. (2012). Fundamentals of transcranial electric and magnetic stimulation dose: definition, selection, and reporting practices. Brain Stimul. 5, 435–453. 10.1016/j.brs.2011.10.001 22305345 PMC3346863

[B23] Pospischil M. Toledo-Rodriguez M. Monier C. Piwkowska Z. Bal T. Frégnac Y.. (2008). Minimal Hodgkin-Huxley type models for different classes of cortical and thalamic neurons. Biol. Cybernet. 99, 427–441. 10.1007/s00422-008-0263-8 19011929

[B24] Rampersad S. Roig-Solvas B. Yarossi M. Kulkarni P. P. Santarnecchi E. Dorval A. D.. (2019). Prospects for transcranial temporal interference stimulation in humans: a computational study. Neuroimage 202:116124. 10.1016/j.neuroimage.2019.116124 31473351 PMC6819277

[B25] Rossi S. Hallett M. Rossini P. M. Pascual-Leone A. Safety of TMS Consensus Group (2009). Safety, ethical considerations, and application guidelines for the use of transcranial magnetic stimulation in clinical practice and research. Clin. Neurophysiol. 120, 2008–2039. 10.1016/j.clinph.2009.08.016 19833552 PMC3260536

[B26] Rossini P. M. Burke D. Chen R. Cohen L. Daskalakis Z. Di Iorio R.. (2015). Non-invasive electrical and magnetic stimulation of the brain, spinal cord, roots and peripheral nerves: basic principles and procedures for routine clinical and research application. An updated report from an IFCN committee. Clin. Neurophysiol. 126, 1071–1107. 10.1016/j.clinph.2015.02.001 25797650 PMC6350257

[B27] Salinas F. Lancaster J. Fox P. (2009). 3D modeling of the total electric field induced by transcranial magnetic stimulation using the boundary element method. Phys. Med. Biol. 54:3631. 10.1088/0031-9155/54/12/002 19458407 PMC5293006

[B28] Sorkhabi M. M. Wendt K. Denison T. (2020). Temporally interfering TMS: focal and dynamic stimulation location, in 2020 42nd Annual International Conference of the IEEE Engineering in Medicine & Biology Society (EMBC) (Montreal, QC: IEEE), 3537–3543. 10.1109/EMBC44109.2020.9176249 PMC7610609 33018767

[B29] Sunshine M. D. Cassará A. M. Neufeld E. Grossman N. Mareci T. H. Otto K. J.. (2021). Restoration of breathing after opioid overdose and spinal cord injury using temporal interference stimulation. Commun. Biol. 4, 1–15. 10.1038/s42003-020-01604-x 33495588 PMC7835220

[B30] Ueno S. Tashiro T. Harada K. (1988). Localized stimulation of neural tissues in the brain by means of a paired configuration of time-varying magnetic fields. J. Appl. Phys. 64, 5862–5864. 10.1063/1.342181

[B31] Yamamoto K. Takiyama Y. Saitoh Y. Sekino M. (2016). Numerical analyses of transcranial magnetic stimulation based on individual brain models by using a scalar-potential finite-difference method. IEEE Trans. Mag. 52, 1–4. 10.1109/TMAG.2016.2519443

[B32] Zaeimbashi M. Khalifa A. Dong C. Wei Y. Cash S. Sun N. (2020). Magnetic temporal interference for noninvasive, high-resolution, and localized deep brain stimulation: concept validation. bioRxiv [Preprint]. 10.1101/2020.07.20.212845

[B33] Zhu X. Li Y. Zheng L. Shao B. Liu X. Li C.. (2019). Multi-point temporal interference stimulation by using each electrode to carry different frequency currents. IEEE Access 7, 168839–168848. 10.1109/ACCESS.2019.2947857

